# Supplementary material for: Validating the British Columbia Perinatal Data Registry: a chart re-abstraction study
Source: BMC Pregnancy Childbirth. 2015 May 27;15:123. doi: 10.1186/s12884-015-0563-7 (PMC4445296; doi:10.1186/s12884-015-0563-7)
Supplement: Additional file 2: — Completion of additional newborn variables (n = 1,142). [file 12884_2015_563_MOESM2_ESM.xls]

Validating the British Columbia Perinatal Data Registry: a chart re-abstraction study

Gillian Frosst, MPH[1]

Jennifer Hutcheon, PhD[1-3]

KS Joseph, MD, PhD[2,3]

Brooke Kinniburgh, MPH[1]

Cathe Johnson, CHIM[1]

Lily Lee (Corresponding Author), RN, MPH[1], West Tower, 3rd Floor, 555 West 12th Avenue, Vancouver, British Columbia, Canada, V5Z 3X7, email: lily.lee@phsa.ca, T: 604-877-2121, F: 604-872-1987

[1]Perinatal Services BC, West Tower, 3rd Floor, 555 West 12th Avenue, Vancouver, British Columbia, Canada, V5Z 3X7

[2]Department of Obstetrics and Gynaecology, University of British Columbia, Shaughnessy Building C420, BC Children's and Women's Hospital, 4500 Oak Street, Vancouver, British Columbia, Canada, V6H 3N1

[3]School of Population and Public Health, University of British Columbia, 2206 East Mall, Vancouver, British Columbia, Canada, V6T 1Z3

Email: Gillian Frosst Gillian.Frosst@interiorhealth.ca; Jennifer Hutcheon jhutcheon@cfri.ca; KS Joseph Ksjoseph@cfri.ca; Brooke Kinniburgh Bkinniburgh@phsa.ca; Cathe Johnson cjohnson@phsa.ca; Lily Lee lily.lee@phsa.ca

Abstract word count (max. 350): 275

Text word count (max. 3,500): 3,259

Number of tables: 6

Number of figures: 0

Number of Additional files: 3 (submitted in a single .xls file)

Background: The British Columbia Perinatal Data Registry (BCPDR) contains individual-level obstetrical and neonatal medical chart data for virtually all births occurring in British Columbia, Canada. The objective of this study was to assess the validity of information in the BCPDR by performing a provincial chart re-abstraction study.

Methods: A two-stage stratified clustered sampling design was employed. Obstetrical facilities were stratified based on geographic location and obstetrical volume. Charts of mothers and newborns with a length of stay of five or more days or transfer to another facility following the delivery were oversampled. A total of 85 maternal and 32 newborn variables were assessed for completeness (percent completion) and validity (sensitivity and specificity for categorical variables, intra-class correlation coefficient [ICC] for continuous variables).

Results: 1,084 maternal and 1,142 newborn charts were abstracted. Mandatory variables such as primary indication for induction and primary indication for cesarean delivery were 100% complete. Some variables such as pre-pregnancy weight were relatively more complete in the re-abstraction as compared with the BCPDR (83.0% vs 76.8%; $p<0.001$). The validity of key surveillance variables was high (e.g., HIV screening completed [sensitivity 98.0%, 95% confidence interval (CI) 97.0-98.8%; specificity 72.3%, 95% CI 60.8-81.9%], induction of labour [sensitivity 93.9%, 95% CI 90.2-96.5%; specificity 98.7%, 95% CI 97.7-99.3%], primary elective cesarean delivery [sensitivity 96.0%, 95% CI 83.8-99.7%; specificity 99.8%, 95% CI 99.4-100.0%], gestational age from newborn examination [ICC 0.99, 95% CI 0.99-0.99]). Examples of variables with lower validity included total admissions prior to delivery episode, maternal smoking status, and timing of breastfeeding initiation.

Conclusion: Many important clinical and population health variables in the BCPDR had excellent validity. Some key variables warrant strengthening through improved definitions, system changes, and abstractor training.

Key words: perinatal care, pregnancy, validation studies, data collection, epidemiology

Background

Perinatal Services BC (PSBC), an agency of the Provincial Health Services Authority (PHSA), has the mandate to improve the capacity and processes of provincial perinatal services through strategic leadership on the full continuum of perinatal care in British Columbia (BC), Canada [1]. PSBC’s mandate is directly supported by the operation and maintenance of the BC Perinatal Data Registry (BCPDR), a provincial database that contains individual-level obstetrical and neonatal medical chart data for virtually all births occurring in BC [2].

The BCPDR has maintained provincial coverage of hospital deliveries and Registered Midwife-attended home births since 2000. The registry collects over 300 data elements for approximately 45,000 births per year. The scope of data spans the antepartum, intrapartum, and postpartum periods and includes information on maternal, fetal, and newborn characteristics. Data from the BCPDR are widely used for surveillance and research purposes and to support health care providers, researchers, and policy makers in their work to improve fetal, neonatal, and maternal health outcomes as well as to enhance the delivery and quality of perinatal care in BC [3-5].

As an administrative database with data entry performed by multiple abstractors at numerous sites across the province, the BCPDR is vulnerable to errors. Data errors can result from incomplete or illegible chart documentation, incorrect data entry, misinterpreted or ambiguous data definitions, and inadequate abstractor training and monitoring [6-8]. To identify and minimize errors, the BCPDR is subject to a rigorous system of on-going quality checks at both the hospital and provincial levels. Small-scale validation studies have provided additional

insights into the reliability of data captured in the registry [9]. However, previous validation studies have typically been one-time projects focusing on a single jurisdiction and/or on select variables and cannot be generalized to provincial-level data or all variables contained in the database. The objective of our study was to perform a large-scale provincial evaluation of BCPDR data elements using expert chart re-abstraction.

Methods

A two-stage stratified clustered sampling design was used to obtain a provincially representative sample of medical charts to undergo re-abstraction. For hospital births, the province's 52 obstetrical facilities were stratified based on geographic location (Vancouver Island, Vancouver Coastal, Fraser, Interior, and Northern Health Authorities, plus BC Women's Hospital [Provincial Health Services Authority]) and obstetrical volume (<1,000, 1,000-2,499, and ≥2,500 deliveries per year). Home births attended by Registered Midwives were sampled independently from two strata based on site of data abstraction. A target sample size of 1,110 charts for each of maternal delivery and baby newborn (neonatal) discharges was based on achieving a precision of +/- 3% assuming (conservatively) an estimated proportion of 50%. The study was powered to detect registry-level differences and was not designed to detect facility-level differences. The sample was allocated across strata using disproportional allocation methodology [10], which increased the sample size for small strata and decreased the sample size for large strata. The sample was equally distributed across all facilities selected within each stratum.

The sampling frame of charts was derived from the BCPDR and included all admission episodes with discharge dates between April 1, 2010 and March 31, 2012. Separate sampling frames were created for maternal and newborn charts. Within each hospital or home birth stratum, half of the charts were selected from the maternal frame and half of the charts were selected from the newborn frame. To ensure adequate sampling of fields pertaining to complex cases, the charts of mothers and babies with a total length of stay of five or more days or transfer to another facility following the delivery or newborn episode were oversampled by 50% of the total sample. Each selected maternal chart was linked to the corresponding newborn chart(s), and vice versa. Linked maternal and newborn charts were re-abstracted including all babies (siblings) from multi-fetal pregnancies. This was done to ensure that the most complete information was re-abstracted for each pregnancy. In some cases, important newborn information was documented in the maternal chart only and vice versa. Pulling both maternal and newborn charts to abstract together helped to generate the most complete information in each of their re-abstracted charts. The final sample included 1,114 mother-baby dyads (or triads) from 17 facilities or births at home.

Re-abstraction was performed by five senior health records personnel with extensive experience working with the BCPDR. Abstractors underwent an additional three-week training period during which inter-rater agreement on an independent sample of maternal and newborn charts was subjectively assessed on an ongoing basis. Differences in abstraction were discussed and consensus was used to determine how to best align responses during the study. Data were entered directly into the existing BCPDR data entry tool using laptops provided by PSBC. Hard error checks (i.e., data entry restrictions based on logic checks) were replaced with soft warnings to permit increased flexibility with data entry. All data fields were re-abstracted with the exception

of diagnosis, procedure, and doctor service fields typically imported from the Canadian Institute for Health Information's Discharge Abstract Database (DAD). Two extra fields designed specifically for this study were included to assess the potential impact of missing chart documentation on the quality of dating ultrasound information. An additional qualitative data collection tool was developed to capture feedback on the usability of individual data input fields from the perspective of the abstractors. The qualitative tool was also used to document information on missing charts and other feedback from the re-abstractors.

Permission to access patient information was obtained from each of the hospitals' data stewards. As a quality assurance project, this study was exempt from Research Ethics Board review under article 2.5 of the TCPS2 (the overarching ethical framework for research involving human participants in Canada including the University of British Columbia BC Children's and Women's Hospital Research Ethics Board). Primary data collection took place from February to April 2013 and was mostly performed on-site to allow access to paper charts. For a small number of facilities with electronic medical records, re-abstraction occurred from a satellite location within the same Health Authority.

The re-abstracted database was linked with the original BCPDR database using a unique numeric identifier assigned to each mother or baby. Analyses were performed using SAS version 9.3 and STATA version 13. Proportions of missing values for each variable were quantified and differences in completion between the re-abstraction database and the BCPDR were tested using a modified Rao-Scott chi-square with a p-value <0.05 considered to be significant [11]. We assessed the validity of variables that were completed in 10 percent or more of deliveries to

ensure that we would be able to estimate validity with reasonable statistical precision. Variables with less than 10 percent completion were typically those that did not apply to all pregnancies (e.g., the variable indicating ‘eligibility for vaginal birth after cesarean’ is only completed for women with a previous cesarean delivery). The re-abstracted data were used as the gold standard, and agreement of the BCPDR data with this gold standard was assessed by calculating sensitivity,specificity, and positive predictive value of categorical variables and the intra-class correlation coefficient (ICC) for continuous variables. Date variables were dichotomized based on completion (completed/missing) and assessed for validity using sensitivity and specificity. Accompanying qualitative data were reviewed and analyzed for themes to identify reasons for discordance between the re-abstracted and original data. The stratified clustered sampling design was incorporated in the analysis using appropriate sampling weights.

Results

Analyses were based on 1,084 maternal charts and 1,142 baby charts. The oversampling criteria resulted in an overrepresentation of multi-fetal pregnancies. Therefore, more newborn charts were re-abstracted compared to maternal charts. In total, 82 maternal and 25 newborn variables met the ≥10% completion criterion and were assessed for validity. As shown in Table 1, the maternal and newborn characteristics in the final weighted cohort were similar to those of all births in the province.

Table 2 presents the completeness of mandatory (i.e., produces a hard error if left blank) variables and other maternal variables routinely used for surveillance. Most variables had high

(≥80%) levels of completion. Examples of variables with lower levels of completion in the BCPDR included pre-pregnancy weight (77%), admission weight (58%), last menstrual period date (68%), and first ultrasound date (71%). Fourteen variables were significantly more complete in the re-abstraction (e.g., height, last menstrual period date). In contrast, Hepatitis B screening results, cervical dilation on admission, and spontaneous labour were significantly more complete in the BCPDR. Completion of first ultrasound date was higher in the re-abstraction database. Further analysis based on the two extra fields indicated that the proportion of missing first ultrasound information would have been reduced by 50% if routine coding instructions had included first ultrasounds between 4-24 weeks (instead of the current instructions to include only ultrasounds between 4-19 weeks). The completion of additional maternal variables is presented in Additional file 2.

Completeness of mandatory and other neonatal variables routinely used for surveillance is shown in Table 3. All variables had high levels of completion in the BCPDR (>90%) with the exception of gestational age from newborn examination (75%). Completion of additional newborn variables is shown in Additional file 2. Variables that were applicable to only a subset of records (e.g., 10 minute Apgar score, surfactant given, resuscitation interventions) had lower levels of completion.

Tables 4 and 5 summarize the prevalence and measures of validity for selected mandatory and other common categorical and continuous maternal variables routinely used for surveillance. Sensitivity, specificity, positive predictive value, and/or ICC were high for most maternal variables. For instance, the BCPDR captured completion of HIV screening with 98.0%

sensitivity (95% confidence interval [CI] 97.0-98.8), and a positive predictive value of 98.3 (95% CI 97.3-99.0). Similarly, the validity of the BCPDR information was high for gestational age at first ultrasound, when available (ICC for gestational age in weeks 0.92, 95% CI 0.90-0.93; ICC for gestational age in days 0.90, 95% CI 0.89-0.92), induction of labour (sensitivity 93.9%, 95% CI 90.2-96.5%; specificity 98.7%, 95% CI 97.7-99.3%), and primary elective cesarean delivery (sensitivity 96.0%, 95% CI 83.8-99.7%; specificity 99.8%, 95% CI 99.4-100.0%). Examples of fields with lower validity included in vitro fertilization (sensitivity 77.5%, 95% CI 61.4-89.2; specificity 98.7%, 95% CI 97.0-99.6; positive predictive value 67.3, 95% CI 43.5-86.0), total prior admissions this pregnancy (ICC 0.59, 95% CI 0.55-0.63), current smoking (sensitivity 63.9, 95% CI 55.6-71.6; specificity 98.2, 95% CI 97.1-99.0-99.6; positive predictive value 81.3 95% CI 70.1-89.6), and positive Hepatitis B screening results (sensitivity 51.1%, 95% CI 18.1-83.4; specificity 99.8, 95% CI 99.4-100.0; positive predictive value 70.7, 95% CI 31.1-95.2). Delivery by family physician, obstetrician, surgeon and midwife showed excellent validity, while delivery by nurse, residents and trainees had lower validity. Sensitivities were high for anterior position in the labour and delivery position variables. The validity of labour and delivery presentation variables was inconsistent with the highest sensitivity noted for vertex presentation. The validity of presentation variables in the BCPDR improved when assessed using a single collapsed ‘breech’ category (labour position sensitivity 75.8%, 95% CI 53.3-91.1; labour presentation specificity 98.9, 95% CI 98.1-99.4; delivery presentation sensitivity 91.2%, 95% CI 82.8-96.4; delivery presentation specificity 99.5, 95% CI 98.8-99.8). Validity varied across indications for induction with the lowest sensitivities noted for fetal compromise, antepartum hemorrhage and other maternal conditions and highest sensitivities for post-dates, pre-labour rupture of membranes diabetes and ‘other’. Validity of primary indications for cesarean delivery

also varied. A relatively large (6.8%) 'other' group was noted for primary indication for operative delivery. Validity of additional maternal variables is provided in Additional file 3.

Tables 6 and 7 show measures of validity for categorical and continuous baby variables, respectively. Basic information such as sex, number of births in pregnancy, head circumference, birth length and discharge weight had excellent validity. Gestational age from newborn examination had a high ICC of 0.99 (95% CI 0.99-0.99). Lower validity was noted for newborn resuscitation variables and breastfeeding. The sensitivity of time of breastfeeding initiation improved using a collapsed ≤24 hours category (81.2%, 95% CI 54.5-95.9), while the specificity decreased (specificity 66.2, 95% CI 55.0-76.3). Sensitivities and positive predictive values were high for exclusive breast milk and lower for formula, breast milk plus formula, and not applicable categories of newborn feeding. Sensitivities and positive predictive values for locations of discharge were high with lowest values observed for discharge to foster care.

Discussion

This provincial chart re-abstraction study showed overall high quality of data contained in the BCPDR with some variation in the completion and validity of certain variables. In general, maternal, antenatal, labour and delivery, drug administration, maternal trauma, postpartum, and newborn information was relatively complete. Within these groups, specific variables related to gestational dating and maternal height and weight appear to be underreported. Many maternal and newborn variables in the BCPDR had high levels of validity where values were available from both the original and re-abstracted records. Lower levels of validity were observed for total

prior admissions during the current pregnancy, position and presentation of baby during labour and delivery, VBAC eligibility, primary indications for induction and cesarean delivery, delivery provider, newborn resuscitation, breastfeeding variables, and postpartum infection.

Variables with lower completion rates mostly required precise measurements or specific dates. Low completion rates in the re-abstracted database may suggest this information is not available in the chart or is not documented in a format that is consistent with current BCPDR data entry specifications (e.g., "high school" instead of the number of school years completed). Low completion variables also tended to be related to sensitive risk factors such as maternal smoking. For other variables, a significantly higher rate of completion in the re-abstracted database suggests the information is available in the chart, but may not be documented in the recommended chart location that is typically reviewed by facility abstractors (e.g., height, weight). It was not surprising that variables in the re-abstraction were generally more complete. Abstractors recruited for this project were asked to thoroughly review all aspects of each chart to retrieve the most accurate information. Also, re-abstraction was carried out without time constraints. In the "real world", facility abstractors may be required to complete the abstraction process within a finite amount of time (e.g., 20 minutes per chart) and as a result, are limited in the time and number of locations they can search for information.

Data entry restrictions for the date and gestational age at first ultrasound fields impacted completion of these variables. The 4-19 weeks restriction was implemented at the time of the BCPDR's inception when determination of gestational age by ultrasound was considered to be

the most accurate prior to 20 weeks. However, recent clinical practice guidelines suggest that ultrasound remains the most accurate method for estimating delivery date up to 23 weeks [12].

Finally, some variables had lower completion rates in the re-abstraction database. Among those with the greatest discrepancy, the lower completion rate for cervical dilation on admission likely resulted from clarifications that arose during the training period about criteria required to abstract this variable. BCPDR guidelines direct abstractors to record the cervical dilation measurement taken within the first hour of admission [13]. However, it was unclear if this definition includes measurements taken in the one hour prior to admission (e.g., during triage) as well as in the one hour after admission. For the purposes of the re-abstraction, abstractors were asked to record only measurements taken in the one hour after admission, which may have increased the number of missing values. For gestational age from newborn examination, the lower completion rate in the re-abstraction may have resulted from different abstractor practices related to gestational age descriptions on the medical chart. For example, care providers may document the gestational age from newborn examination as "term" on the medical chart. Some site abstractors may have translated this into a gestational age (e.g., 40 weeks) for the purposes of the BCPDR, whereas the re-abstraction staff would have left the field blank.

Potential explanations for disagreements between the two databases were highly variable dependent. Incorrect documentation of position and presentation has been identified previously through routine data quality reviews. To address this known issue, a PSBC Bulletin was issued in 2011 to provide clearer guidance to abstractors for determining this information from the chart [14]. This educational strategy was implemented part-way through the re-abstraction study

period, which may account for some of the disagreement observed. For cesarean delivery indications, feedback from abstractors highlighted that several indications may be provided for a delivery, requiring the abstractor to determine the most important (primary) indication for purposes of data entry. The same also applies to the primary indication for induction. Discordance within primary indication for operative delivery has also been reported previously and attributed, in part, to the absence of a specific place for consistent documentation in the chart and ambiguity of indication categories in the BCPDR [15]. Furthermore, the relatively large proportion of records with an 'other' primary indication for operative delivery suggests that existing response options may not be appropriate for current practice. The discordance in other variables such as postpartum infection may also be explained by the absence of a specific place for documentation on the provincial perinatal forms.

The lower sensitivity for "unknown time of stillbirth" likely reflects the larger clinical challenge of determining time of fetal demise in utero. Lower validity across the newborn resuscitation variables may have resulted from lack of clarity in the abstractor guidelines regarding definitions of resuscitation, ventilation, invasive and non-invasive CPAP, as well as time and place of intervention. These definitions have since been expanded upon and clarified in an updated version of the PSBC Reference Manual [16]. The lower sensitivity of breastfeeding initiation was likely impacted by the use of multiple versions of the Newborn Clinical Path record, which contained different breastfeeding interval categories, across the province. Qualitative feedback from the abstractors also indicated challenges with determining precise time of breastfeeding initiation where this was not clearly documented in the charts. This was reflected in the relatively large 'unknown' categories for both breastfeeding variables. The moderate ICC noted for gravida

was due to a small number of identified typos in the re-abstraction study and should be interpreted as negligible for the purposes of this evaluation. The discrepancy for total prior admissions during the current pregnancy may have been due to reduced access to medical charts from prior admissions during the re-abstraction and should also be interpreted with caution. Finally, site abstractors were likely more familiar with names and designations of local health care providers thereby contributing to disagreement for the delivery provider variable.

The Niday Perinatal Database (NPD) in Ontario, Canada, has undergone a similar quality assurance evaluation using chart re-abstraction to determine the reliability, completeness, and comprehensiveness of provincial perinatal data. The findings for most data fields between the NPD and the BCPDR were similar. Examples of variables with different findings include gestational age at delivery and birth weight, both of which had excellent validity in the BCPDR but had poor agreement in the NPD. In contrast, the validities of "breech" and "dystocia" as indications for cesarean delivery were lower in the BCPDR compared to the NPD [17]. Although we did not assess the validity of diagnosis, procedure, and doctor service fields imported into the BCPDR from the DAD, clinical coding practices of hospitals contributing to the DAD are reviewed on an on-going basis [e.g., 18]. Validation of key perinatal fields captured by the DAD has also occurred through comparison to another provincial perinatal database in Canada, the Nova Scotia Atlee Perinatal Database [19].

Strengths and Limitations

Key strengths of this study include a large sample size, provincial representation of hospital and home births in BC, and inclusion of many variables across the perinatal continuum. Furthermore, the mixed methods approach allowed us not only to quantify discordance and validity measures, but also to elucidate potential reasons for differential variable performance using qualitative feedback. For the purposes of this validation study, information documented in the medical chart was assumed to be accurate. However, the findings are limited by the absence of a true gold standard with which to compare BC's perinatal data registry. We compared the registry data against data obtained from abstractors who were highly experienced in obstetrical coding, routinely worked with BCPDR data, and underwent an extensive training period to clarify ambiguities in the abstractor guidelines prior to primary data collection. Electronic medical records with data entered by care providers at the point of care may help to increase the accuracy of the BCPDR in the future; however, until such time as a provincially-integrated system is available, chart abstraction is required. The results presented here were derived using sampling weights based on a sampling frame of designated obstetrical facilities. Thus, the results may not reflect the small number of charts for births that occurred in non-obstetrical facilities during the study period. High ICCs for continuous variables should be interpreted with caution as they were calculated after excluding charts with missing values. Finally, the sample of charts included in the study was too small to validate variables that represent low prevalence interventions such as some methods of induction and augmentation, conditions such as blood transfusions and severe maternal and newborn morbidity, and most maternal risk factors (e.g., gestational hypertension, gestational diabetes, antepartum hemorrhage, and congenital anomalies in prior pregnancy).

Conclusion

Overall, the validity of the BCPDR data elements was very good with some variation noted for specific variables. Most common clinical and population health variables had excellent validity, supporting the use of the registry data for public health surveillance and research. Some variables need to be strengthened through improved definitions, system changes and enhanced abstractor training. This study contributes valuable information that will help to improve the quality of BCPDR data elements and thereby help in the creation of a core dataset to be integrated into the upcoming registry redevelopment. This information will also inform decisions to keep or delete certain variables and will provide the evidence base for initiating the important dialogue necessary for modifying suboptimal - yet highly critical – variables for the purposes of surveillance, monitoring, evaluation or research.

List of Abbreviations

BC – British Columbia

BCPDR – BC Perinatal Data Registry

95% CI – 95% confidence interval

ICC – Intra-Class Correlation Coefficient

PSBC – Perinatal Services BC

PHSA – Provincial Health Services Authority

Competing Interests

None declared.

Authors' contributions

GF, JH, KSJ, BK, LL and CJ participated in the design and implementation of the study. GF coordinated the study, led the data collection process, performed the analysis, and drafted the manuscript. JH provided additional analytic support. JH, KSJ, BK, LL and CJ revised the manuscript for intellectual content. GF, JH, KSJ, BK, LL and CJ approved the final manuscript.

Authors' information

None.

Acknowledgments:

We thank Perinatal Services BC staff Laura Bailey, Susan Barker, Linda Lee, Lisa Miyazaki, and Elaine Worden for their assistance with chart abstractions and Kenny Der for co-ordinating access to the charts at each of the hospitals.

References

1. Provincial Health Services Authority: **Perinatal Services BC.** [http://www.phsa.ca/AgenciesAndServices/Agencies/perinatalservicesbc.htm].

2. Perinatal Services BC: **Data registry.** [http://www.perinatalservicesbc.ca/DataAndSurveillance/DataRegistry/default.htm].

3. Grzybowski S, Stoll K, Kornelsen J: **The outcomes of perinatal surgical services in rural British Columbia: a population-based study.** *Can J Rural Med* 2013 Fall;**18(4):**123-9.

4. Marquette GP, Hutcheon JA, Lee L: **Predicting the spontaneous onset of labour in post-date pregnancies: a population-based retrospective cohort study.** *J Obstet Gynaecol Can* 2014 May;**36(5):**391-9.

5. Mehrabadi A, Hutcheon JA, Lee L, Kramer MS, Liston RM, Joseph KS: **Epidemiological investigation of a temporal increase in atonic postpartum haemorrhage: a population-based retrospective cohort study.** *BJOG* 2013 Jun;**120(7):**853-62.

6. Bell JC, Ford JB, Cameron CA, Roberts CL: **The accuracy of population health data for monitoring trends and outcomes among women with diabetes in pregnancy.** *Diabetes Res Clin Pract* 2008 Jul;**81(1):**105-9.

7. Bradford HM, Cárdenas V, Camacho-Carr K, Lydon-Rochelle MT: **Accuracy of birth certificate and hospital discharge data: a certified nurse-midwife and physician comparison.** *Matern Child Health J* 2007 Nov;**11(6):**540-8.

8. Reeves MJ, Mullard AJ, Wehner S**: Inter-rater reliability of data elements from a prototype of the Paul Cloverdell National Acute Stroke Registry.** *BMC* 2008 **8:**19.

9. MacIntyre E, Linnegar M, Lencar C, Brauer M, Demers P, Ostry A: **Perinatal database registry chart abstraction validation report.** Vancouver (BC): University of British Columbia; 2006.

10. Statistics Canada: *Survey methods and practices.* Ottawa: Minister of Industry; 2010

11. Scott A: **Rao-Scott corrections and their impact.** In *Proceedings of the Survey Research Methods Section, ASA*: *2007.* [https://www.amstat.org/sections/srms/proceedings/y2007/Files/JSM2007-000874.pdf]

12. Butt K, Lim K: **Determination of gestational age by ultrasound.** *J Obstet Gynaecol Can* 2014 Feb; **36(2):**171-181.

13. Perinatal Services BC: **British Columbia Perinatal Data Registry Reference Manual, Version 6.0.** Vancouver (BC): Perinatal Services BC; 2008.

14. Perinatal Services BC: **Spotlight on presentation and position.** *PSBC Bulletin* 2011 Oct;**4(3):**5.

15. Perinatal Services BC: **Validation of the indications for cesarean delivery in the British Columbia Perinatal Data Registry: A Perinatal Services BC Surveillance Special Report.** 2012 Apr;**1(5):**10.

16. Perinatal Services BC: **British Columbia Perinatal Data Registry Reference Manual, Version 6.01.** Vancouver (BC): Perinatal Services BC; 2014.

17. Dunn S, Bottomley J, Ali A, Walker M: **2008 Niday Perinatal Database quality audit: report of a quality assurance project**. *Chronic Dis Inj Can*. 2011 Dec;**32(1):**32-42.

18. Canadian Institute for Health Information (CIHI): **CIHI Data Quality Study of the 2009-2010 Discharge Abstract Database**. Ottawa (ON): CIHI; 2012.

19. Joseph KS, Fahey J: **Validation of perinatal data in the Discharge Abstract Database of the Canadian Institute for Health Information**. *Chronic Dis Can*. 2009;**29(3):**96-100.

Table 1. Maternal and neonatal characteristics of deliveries sampled for chart re-abstraction, British Columbia, 2010-2012.

Characteristic		Unweighted Sample mean± SD or n(%)	Weighted sample mean± SD or n(%)	British Columbia population mean± SD or n(%)
Maternal		1,084	87,266	87,318
	Maternal age (years)	30.6± 5.8	30.6± 5.8	30.3± 5.5
	Parity (nulliparous)	551 (50.8)	42,775 (49.0)	40,835 (46.8)
	Pre-pregnancy Body Mass Index (kg/m^2)	25.5± 5.8	24.6± 5.4	24.4± 5.3†
	Smoking during pregnancy	148 (13.7)	9,372 (10.7)	7,305 (8.4)
	Gestational age at delivery (weeks)	37.8± 3.2	38.4± 2.7	38.5± 2.5
Newborn	N	1,142	88,720	88,759
	Birthweight (g)	3045± 710	3211± 553	3360± 631
	Sex (male)	574 (50.3)	42,715 (48.1)	45,393 (51.2)
	Multiple birth (twin or higher order)	108 (9.5)	3,410 (3.9)	2,864 (3.2)

†among women with available pre-pregnancy BMI

Table 2. Completion of mandatory and other maternal variables routinely used for surveillance (n=1,084)

Variable Name	% complete in reabstraction[a]	% complete in BCPDR[a]	p-value if <0.05
**Antenatal information**			
Maternal date of birth[b]	100.0	100.0	
Pre-pregnancy weight	83.0	76.8	<0.001
Admission weight	66.9	58.3	<0.001
Height	82.4	71.2	<0.001
Gravida[b]	100.0	100.0	
Last menstrual period date	73.9	68.1	<.0001
First ultrasound date	83.5	71.1	<.0001
Gestational age at first ultrasound, in weeks	83.5	71.8	<.0001
Gestational age at first ultrasound, in days (+0-6)	78.8	67.4	<.0001
In vitro fertilization[b]	100.0	100.0	
Total prior admissions this pregnancy[b]	100.0	100.0	
Smoking status	62.7	49.8	<.0001
School years completed	32.9	31.6	<.0001
Number of antenatal visits	96.9	90.4	<.0001
Blood type[b]	100.0	100.0	
Maternal serum screening offered[b]	100.0	100.0	
Hepatitis B screening completed[b]	100.0	100.0	
Hepatitis B screening results[d]	95.0	95.1	<.0001
Group B Strep screening completed[b]	100.0	100.0	
Group B Strep screening results[d]	83.6	82.6	<.0001
HIV screening completed[b]	100.0	100.0	
**Labour and delivery information**			
Cervical dilation on admission	53.9	65.4	<.0001
Vaginal birth after cesarean delivery (VBAC) eligible[d]	16.4	16.4	
VBAC attempted[b]	100.0	100.0	
Labour type - Spontaneous	59.9	60.8	<.0001
Labour type - Augmented[c]	35.9	33.5	<.0001
Labour type - Induced	23.3	22.9	<.0001

Labour type - None	16.9	16.3	<.0001
Labour type - Unknown	0.0	0.0	
Primary indication for induction[b,c]	100.0	100.0	
Labour position[b,c]	100.0	100.0	
Labour presentation[b,c]	100.0	100.0	
Delivery position[b,c]	100.0	100.0	
Delivery presentation[b,c]	100.0	100.0	
Primary indication for operative delivery[b,c]	100.0	100.0	
Cesarean delivery type[b,c]	100.0	100.0	
Cesarean incision[b,c]	100.0	100.0	
Baby delivery date[b,c]	100.0	100.0	
Baby delivery time[b,c]	100.0	100.0	
Delivery provider (delivered by)[b,c]	100.0	100.0	
Rh immunoglobulin postpartum eligible[b]	100.0	100.0	
Postpartum infection[b]	100.0	100.0	

[a]Proportions based on weighted data
[b]Mandatory fields
[c]Multi-fetal pregnancies included one record per baby delivered (n=1,129)
[d]Variable only applies to subset of population

Table 3. Completion of mandatory and other newborn variables routinely used for surveillance (n=1,142)

Variable Name	% complete in reabstraction[a]	% complete in BCPDR[a]	p-value if <0.05
**Newborn information**			
Date of birth[b]	100.0	100.0	
Sex[b]	100.0	100.0	
Number of births in pregnancy[b]	100.0	100.0	
Gestational age from newborn examination	66.8	74.7	<.0001
Gestational age from maternal chart	99.2	99.5	
5 minute Apgar score	100.0	100.0	
Head circumference	98.5	99.0	<.0001
Length	98.7	98.8	<.0001
First temperature	89.5	90.8	<.0001
Stillbirth timing	100.0	100.0	
Resuscitative drugs[b]	100.0	100.0	
Stabilization - Oxygen days[b]	100.0	100.0	
Stabilization - Ventilator days[b]	100.0	100.0	
Stabilization - CPAP days[b,c]	100.0	100.0	
TPN days[b,c]	100.0	100.0	
Breastfeeding initiation[b]	100.0	100.0	
Newborn feeding[b]	100.0	100.0	
Discharge weight	92.3	92.1	<.0001
Discharge to[b]	100.0	100.0	

[a]Proportions based on weighted data
[b]Mandatory fields
[c]CPAP refers to continuous positive airway pressure and TPN denotes total parenteral nutrition.

Table 4. Validity of selected mandatory and other common categorical variables from maternal charts (n=1,084[d])

Variable Name	Prevalence[a,b]	Sensitivity (95% CI)[a]	Specificity (95% CI)[a]	Positive predictive value (95% CI)
**Antenatal info**				
Maternal date of birth (Completed)[c]	100.0	100.0 (-)	-	100.0 (-)
Last menstrual period date (Completed)[c]	73.9	84.4 (81.6 - 87.0)	78.3 (72.1 - 83.6)	91.7 (89.4 - 93.6)
First ultrasound date (Completed)[c]	83.5	83.9 (78.9 - 88.2)	93.8 (89.4 - 96.7)	98.6 (97.4 - 99.3)
In vitro fertilization (Yes)	3.3	77.5 (61.4 - 89.2)	98.7 (97.0 - 99.6)	67.3 (43.5 - 86.0)
Smoking status (current)	10.7	63.9 (55.6 - 71.6)	98.2 (97.1 - 99.0)	81.3 (70.1 - 89.6)
Maternal serum screening offered (Yes)	81.3	91.4 (88.6 - 93.8)	80.9 (72.5 - 87.6)	95.4 (92.7 - 97.3)
Hepatitis B screening completed (Yes)	95.0	98.2 (97.2 - 98.9)	64.0 (51.1 - 75.5)	98.1 (96.7 - 99.0)
Positive	0.7	51.1 (18.1 - 83.4)	99.8 (99.4 - 100.0)	70.7 (31.1 - 95.2)
Group B Strep screening completed (Yes)	83.6	93.0 (91.1 - 94.7)	70.7 (63.3 - 77.4)	94.2 (91.7 - 96.1)
Positive	23.8	91.1 (81.9 - 96.6)	97.8 (95.3 - 99.2)	92.8 (86.6 - 96.8)
HIV screening completed (Yes)	94.3	98.0 (97.0 - 98.8)	72.3 (60.8 - 81.9)	98.3 (97.3 - 99.0)
**Labour and delivery information**				
Vaginal birth after cesarean delivery (VBAC) eligible	9.2	74.2 (63.4 - 83.2)	98.0 (96.9 - 98.8)	79.1 (68.7 - 87.3)
VBAC attempted	4.7	85.1 (64.4 - 96.3)	100.0 (99.6 - 100.0)	100.0 (90.9 -100.0)
Labour type - Spontaneous	59.9	97.9 (96.4 - 98.8)	94.4 (91.2 - 96.7)	96.3 (94.6 - 97.6)
Labour type - Augmented	35.9	81.8 (75.9 - 86.8)	93.5 (90.9 - 95.6)	87.7 (83.2 - 91.3)
Labour type - Induced	23.3	93.9 (90.2 - 96.5)	98.7 (97.7 - 99.3)	95.5 (92.4 - 97.7)
Labour type - None	16.9	94.0 (89.9 - 96.8)	99.5 (98.8 - 99.8)	97.3 (94.0 - 99.1)
Primary indication for				

induction				
Post dates	6.3	87.1 (76.2 - 94.3)	99.0 (97.3 - 99.8)	85.7 (70.9 - 94.8)
Pre-labour rupture of membranes	5.7	90.7 (76.2 - 97.8)	98.7 (97.8 - 99.3)	80.9 (69.8 - 89.4)
Fetal compromise	2.0	40.1 (22.4 - 59.8)	99.2 (98.5 - 99.6)	50.1 (29.3 - 71.0)
Other maternal condition	2.7	28.9 (8.5 - 58.7)	99.5 (98.5 - 99.9)	63.1 (18.2 - 95.0)
Logistics	0.1	100.0 (-)	100.0 (-)	100.0 (-)
Fetal demise	0.2	100.0 (-)	100.0 (-)	100.0 (-)
Hypertension in pregnancy	3.1	73.2 (59.2 - 84.6)	100.0 (99.6 - 100.0)	99.0 (89.3 - 100.0)
Antepartum hemorrhage	0.2	14.1 (0.0 - 90.4)	100.0 (-)	100.0 (-)
Diabetes	0.8	94.7 (63.7 - 100.0)	99.6 (99.0 - 99.9)	65.3 (37.1 - 87.3)
Other	1.6	83.2 (59.2 - 96.1)	98.3 (97.3 - 99.0)	44.6 (27.6 - 62.6)
Unknown	0.4	-	99.7 (98.5 - 100.0)	-
Delivery presentation				
Breech/NOS	3.3	62.8 (47.9 - 76.2)	98.9 (98.1 - 99.4)	66.3 (51.7 - 78.9)
Frank breech	0.7	62.8 (30.1 - 88.6)	99.6 (99.0 - 99.9)	51.1 (20.8 - 80.8)
Footling breech	1.3	70.0 (38.2 - 91.8)	99.6 (99.0 - 99.9)	71.4 (49.0 - 88.1)
Complete breech	0.2	-	99.9 (99.4 - 100.0)	-
Incomplete breech	0.0	-	99.9 (99.4 - 100.0)	-
Vertex	89.8	98.0 (96.8 - 98.8)	65.7 (51.5 - 78.1)	96.2 (92.5 - 98.4)
Transverse	1.1	54.0 (26.3 - 80.0)	99.6 (98.6 - 99.9)	57.9 (27.3 - 84.6)
Other	0.0	-	100.0 (-)	-
Unknown	3.5	13.7 (4.7 - 29.0)	98.5 (96.3 - 99.6)	25.4 (9.5 - 48.2)
Primary indication for operative delivery				
Breech	3.0	75.4 (58.5 - 88.0)	99.9 (99.5 - 100.0)	95.3 (83.1 - 99.5)
Dystocia/CPD	7.3	50.9 (40.7 - 61.0)	99.6 (99.0 - 99.9)	90.5 (80.7 - 96.4)

Nonreassuring Fetal Heart Rate	5.9	85.9 (76.3 - 92.7)	98.7 (97.6 - 99.4)	81.1 (71.0 - 88.9)
Repeat Cesarean Section	4.4	79.3 (64.6 - 89.9)	95.2 (92.5 - 97.1)	43.0 (25.6 - 61.7)
Abruptio Placenta	0.3	87.3 ([e])	99.9 (99.5 - 100.0)	74.0 (27.0 - 98.1)
Placenta Previa	0.9	94.4 (69.6 - 99.9)	100.0 (99.6 - 100.0)	96.3 (69.5 - 100.0)
Malposition/Malpresentation	1.8	67.3 (42.4 - 86.6)	98.4 (97.4 - 99.0)	42.7 (20.2 - 67.7)
Active Herpes	0.0	100.0 (-)	100.0 (-)	100.0 (-)
VBAC Declined/Maternal Request	5.4	36.4 (17.6 - 58.8)	99.0 (98.3 - 99.5)	68.4 (48.6 - 84.3)
Unknown	0.0	-	99.8 (99.4 - 100.0)	-
Other	6.8	71.5 (60.3 - 81.2)	96.7 (95.0 - 98.0)	61.7 (43.4 - 77.8)
Cesarean delivery type				
Primary Elective	3.5	96.0 (83.8 - 99.7)	99.8 (99.4 - 100.0)	95.3 (82.3 - 99.6)
Primary Emergency	17.7	99.1 (97.1 - 99.8)	99.8 (99.2 - 100.0)	99.2 (97.3 - 99.9)
Repeat Elective	7.3	94.9 (86.7 - 98.8)	99.2 (98.0 - 99.8)	90.7 (76.0 - 97.9)
Repeat Emergency	7.3	90.2 (78.9 - 96.7)	99.6 (99.0 - 99.9)	94.7 (87.9 - 98.3)
Baby delivery date (Completed)[c]	100.0	100.0 (-)	-	100.0 (-)
Baby delivery time (Completed)[c]	100.0	100.0 (-)	-	100.0 (99.6 -100.0)
Delivery provider (Delivered by)[d,e]				
Family Physician	33.6	82.9 (67.0 - 93.2)	96.2 (93.6 - 98.0)	91.8 (86.9 - 95.3)
Obstetrician	46.3	96.9 (95.2 - 98.2)	89.6 (85.6 - 92.8)	88.9 (84.9 - 92.2)
Surgeon	0.9	100.0 (-)	100.0 (-)	100.0 (-)
Midwife	9.0	97.4 (92.6 - 99.4)	99.4 (98.8 - 99.8)	94.5 (88.8 - 97.9)
Nurse	2.5	64.9 (35.7 - 87.6)	99.8 (99.3 - 100.0)	89.4 (61.7 - 99.3)
Rh immunoglobulin postpartum eligible (Yes)	7.2	89.6 (80.5 - 95.4)	99.8 (99.3 - 100.0)	97.0 (89.8 - 99.6)
Postpartum infection (Yes)	0.9	65.3 (37.1 - 87.3)	99.2 (98.4 - 99.6)	40.8 (21.4 - 62.6)

1 [a]Prevalence and measures of validity based on weighted data

[b]Prevalence based on re-abstraction
[c]Variable was dichotomized (completed/missing); completed values were assumed to be equal
[d]Multi-fetal pregnancies included one record per baby delivered (n=1,129)
[e]Additional response options shown in Additional file 3

Table 5. Agreement measures for continuous variables from maternal charts (n=1,084)

Variable Name	ICC (95% CI)[a,b]
**Maternal information**	
Pre-pregnancy weight	0.97 (0.96 -0.97)
Admission weight	0.94 (0.94 -0.95)
Height	0.90 (0.89 -0.92)
**Antenatal information**	
Gravida	0.76 (0.73 -0.78)
Previous term deliveries	0.99 (0.98 -0.99)
Previous preterm deliveries	0.86 (0.85 -0.88)
Previous spontaneous abortions	0.92 (0.91 -0.93)
Previous therapeutic abortions	0.90 (0.88 -0.91)
Previous cesarean deliveries	0.99 (0.99 -0.99)
Previous vaginal deliveries	0.99 (0.99 -0.99)
Number of living children	0.98 (0.98 -0.99)
Third trimester hemoglobin	0.85 (0.83 -0.87)
Gestational age at first ultrasound, in weeks	0.92 (0.90 -0.93)
Gestational age at first ultrasound, in days	0.90 (0.89 -0.92)
Total prior admissions this pregnancy	0.59 (0.55 -0.63)
School years completed	0.92 (0.90 -0.94)
Number of antenatal visits	0.91 (0.90 -0.92)
**Labour and delivery information**	
Cervical dilation on admission	0.88 (0.86 -0.90)
Cervical dilation prior to cesarean delivery	0.96 (0.95 -0.97)
Postpartum hemoglobin value	0.98 (0.98 -0.98)

[a]Measure of validity based on weighted data
[b]ICC calculation based on records that were complete in both the BCPDR and reabstraction database; does not account for disagreement due to missing values

Table 6. Validity of common categorical variables from newborn charts (n=1,142)

Variable Name	Prevalence[a,b]	Sensitivity (95% CI)[a]	Specificity (95% CI)[a]	Positive predictive value (95% CI)
**Mother information**				
Mother's date of birth (Completed)[c]	99.3	100.0 (-)	-	99.3 (98.6 - 99.7)
**Newborn information**				
Date of birth (Yes)[c]	100	100.0 (-)	-	100.0 (-)
Sex				
Female	51.7	99.9 (99.2 - 100.0)	99.7 (98.9 - 100.0)	99.7 (98.9 - 100.0)
Male	48.1	99.7 (98.9 - 100.0)	99.5 (98.6 - 99.9)	99.5 (98.5 - 99.9)
Number of births in pregnancy				
1	94.9	100.0 (-)	100.0 (-)	100.0 (-)
2	5.1	100.0 (-)	100.0 (-)	100.0 (-)
Stillbirth timing				
N/A (live birth)	98.5	100.0 (-)	100.0 (-)	100.0 (-)
Prior to onset of labour	0.5	85.3 (26.6 - 100.0)	99.7 (99.2 - 99.9)	59.8 (14.5 - 94.6)
After onset of labour	0	-	99.9 (99.4 - 100.0)	-
Unknown time of stillbirth	0.9	53.2 ([d])	99.9 (99.5 - 100.0)	86.2 ([d])
Meconium present	18.3	76.9 (62.4 - 87.8)	97.6 (96.5 - 98.5)	87.9 (81.9 - 92.5)
Suction - Oropharynx	18.8	58.7 (40.6 - 75.1)	97.4 (93.9 - 99.2)	84.1 (76.3 - 90.2)
Resuscitative drugs	1.6	64.5 (48.8 - 78.2)	99.8 (99.3 - 100.0)	81.7 (65.8 - 92.3)
Resuscitation - Oxygen	12.9	74.8 (61.4 - 85.5)	99.3 (98.6 - 99.8)	94.3 (86.7 - 98.3)
Breastfeeding initiation				
0 to ≤1hr	45.7	70.9 (61.6 - 79.0)	80.9 (72.9 - 87.3)	75.7 (69.4 - 81.3)
>1 to ≤24hrs	42.6	52.6 (28.7 - 75.7)	81.8 (77.8 - 85.4)	68.2 (61.9 - 74.0)

>24hrs	2.9	18.1 (6.8 - 35.7)	98.8 (98.0 - 99.4)	31.7 (20.2 - 45.1)
Unknown	4.1	46.9 (31.0 - 63.3)	82.9 (55.1 - 97.0)	10.4 (5.6 - 17.4)
N/A (did not breastfeed, died, or stillborn)	4.8	68.1 (60.2 - 75.3)	98.7 (97.8 - 99.3)	73.1 (63.8 - 81.1)
Newborn feeding				
Breast milk	63.4	90.7 (87.9 - 93.0)	78.8 (74.7 - 82.5)	88.1 (85.2 - 90.7)
Formula	2.4	59.6 (42.9 - 74.8)	98.8 (97.9 - 99.3)	55.0 (41.0 - 68.4)
Breast milk & formula	30.6	72.7 (67.4 - 77.7)	90.5 (88.1 - 92.5)	77.1 (72.6 - 81.2)
Unknown	2.3	75.8 (67.0 - 83.3)	100.0 (99.6 - 100.0)	98.2 (90.2 - 100.0)
N/A (transferred to another hospital, died, or stillborn)	1.2	73.4 ([d])	99.5 (98.9 - 99.8)	63.1 (51.4 - 73.8)
Discharge to				
Home	95.5	99.7 (99.1 - 100.0)	94.6 (82.6 - 99.2)	99.7 (99.1 - 100.0)
Other Hospital	2.3	100.0 (-)	100.0 (-)	100.0 (-)
Adoption	0	100.0 (-)	99.8 (99.3 - 100.0)	3.0 ([d])
Foster	0.6	69.2 (29.6 - 94.7)	100.0 (99.6 - 100.0)	90.4 (59.9 - 99.6)
Death/Stillbirth	1.5	100.0 (-)	100.0 (-)	100.0 (-)
Unknown	0.1	-	100.0 (-)	-

[a]Prevalence and measures of validity based on weighted data
[b]Prevalence based on re-abstraction
[c]Variables were dichotomized (completed/missing); completed values were assumed to be equal
[d]95% confidence interval not estimable

Table 7. Agreement measures for common continuous variables from newborn charts (n=1,142)

**Variable Name**	**ICC (95% CI)[a,b]**
**Newborn information**	
1 minute Apgar score	0.99 (0.99-0.99)
5 minute Apgar score	0.99 (0.99-0.99)
10 minute Apgar score	1.0 (1.0-1.0)
Head circumference	0.96 (0.95 -0.96)
Length	0.98 (0.98 -0.98)
First temperature	0.86 (0.84-0.87)
Gestational age from newborn examination	0.99 (0.99-0.99)
Gestational age from maternal chart	1.0 (0.99-1.0)
Stabilization - Oxygen days	0.88 (0.87-0.90)
Stabilization - Ventilator days	0.86 (0.85-0.88)
Stabilization - CPAP days[c]	0.95 (0.95-0.96)
TPN days[c]	0.98 (0.98 -0.98)
Discharge weight	0.99 (0.98 -0.99)

[a]Measure of validity based on weighted data
[b]ICC calculation based on records that were complete in both the BCPDR and reabstraction database; does not account for disagreement due to missing values
[c]CPAP refers to continuous positive airway pressure and TPN denotes total parenteral nutrition

Additional files

File name: Additional file 1

File format: MS Excel (.xls)

Title: Completion of additional maternal variables (n=1,084)

Description: Appendix to Table 1 in manuscript

File name: Additional file 2

File format: MS Excel (.xls)

Title: Completion of additional newborn variables (n=1,142)

Description: Appendix to Table 2 in manuscript

File name: Additional file 3

File format: MS Excel (.xls)

Title: Validity of additional common categorical variables from maternal charts (n=1,084)

Description: Appendix to Table 3 in manuscript
